# Lulworthinone: In Vitro Mode of Action Investigation of an Antibacterial Dimeric Naphthopyrone Isolated from a Marine Fungus

**DOI:** 10.3390/md20050277

**Published:** 2022-04-21

**Authors:** Eric Juskewitz, Ekaterina Mishchenko, Vishesh K. Dubey, Marte Jenssen, Martin Jakubec, Philip Rainsford, Johan Isaksson, Jeanette H. Andersen, Johanna U. Ericson

**Affiliations:** 1 Research Group for Host Microbe Interactions, Department of Medical Biology, Faculty of Health Sciences, UiT the Arctic University of Norway, 9019 Tromsø, Norway; ekaterina.mischenko@uit.no (E.M.); vishesh.k.dubey@uit.no (V.K.D.); 2 Marbio, The Norwegian College of Fishery Science, Faculty of Biosciences, Fisheries and Economics, UiT the Arctic University of Norway, 9019 Tromsø, Norway; marte.jenssen@uit.no (M.J.); jeanette.andersen@uit.no (J.H.A.); 3 Department of Chemistry, Faculty of Science and Technology, UiT the Arctic University of Norway, 9019 Tromsø, Norway; martin.jakubec@uit.no (M.J.); philip.rainsford@uit.no (P.R.); johan.isaksson@uit.no (J.I.)

**Keywords:** marine natural product, antimicrobial agents, mode of action, *B. subtilis*, MRSA, FtsZ, colloidal aggregate

## Abstract

Treatment options for infections caused by antimicrobial-resistant bacteria are rendered ineffective, and drug alternatives are needed—either from new chemical classes or drugs with new modes of action. Historically, natural products have been important contributors to drug discovery. In a recent study, the dimeric naphthopyrone lulworthinone produced by an obligate marine fungus in the family *Lulworthiaceae* was discovered. The observed potent antibacterial activity against Gram-positive bacteria, including several clinical methicillin-resistant *Staphylococcus aureus* (MRSA) isolates, prompted this follow-up mode of action investigation. This paper aimed to characterize the antibacterial mode of action (MOA) of lulworthinone by combining in vitro assays, NMR experiments and microscopy. The results point to a MOA targeting the bacterial membrane, leading to improper cell division. Treatment with lulworthinone induced an upregulation of genes responding to cell envelope stress in *Bacillus subtilis*. Analysis of the membrane integrity and membrane potential indicated that lulworthinone targets the bacterial membrane without destroying it. This was supported by NMR experiments using artificial lipid bilayers. Fluorescence microscopy revealed that lulworthinone affects cell morphology and impedes the localization of the cell division protein FtsZ. Surface plasmon resonance and dynamic light scattering assays showed that this activity is linked with the compound‘s ability to form colloidal aggregates. Antibacterial agents acting at cell membranes are of special interest, as the development of bacterial resistance to such compounds is deemed more difficult to occur.

## 1. Introduction

Antimicrobial-resistant bacterial pathogens have emerged as a serious threat to public health, and there is an urgent need for new antibiotics. In 2019, infections caused by antimicrobial-resistant (AMR) bacteria were the third leading cause of death. Patients infected by *Staphylococcus aureus* were 64% more likely to die if the strain was methicillinresistant than if it was susceptible. As a result, methicillin-resistant *S. aureus* (MRSA) alone killed over 100,000 patients globally in 2019 [1]. Thus, the World Health Organisation (WHO) has declared MRSA as one of their priority pathogens to develop treatments against. Since AMR mechanisms are known to evolve and protect against related drug iterations, there is an urgent need for compounds with either a new mode of action (MOA) or from new chemical classes. Currently, 32 antibiotics targeting the WHO priority pathogens are under development. However, only six of them fulfil typical criteria for innovation (absence of cross-resistance, new chemical class, new target or new mode of action) [2,3]. The last truly new antibiotic class discovered were acid lipopeptides in 1987 [4].

Still, unexplored parts of nature can provide new molecules with novel antibacterial properties. Bioprospecting has the potential to supply the drug development pipeline with new compounds. Through history, natural products have contributed the most to the development of drugs in clinical use [5]. Either they contain the antibacterial activity themselves (e.g., aminoglycosides, β-lactams, macrolides, tetracyclines) [6] or their molecule scaffolds have been adapted for drug development [7]. The focus on marine bioprospecting has increased in the last decades. Due to the dilution processes occurring in seawater, the antimicrobial compounds produced by marine organisms should be highly potent in order to be effective against their targets.

**Figure 1:**
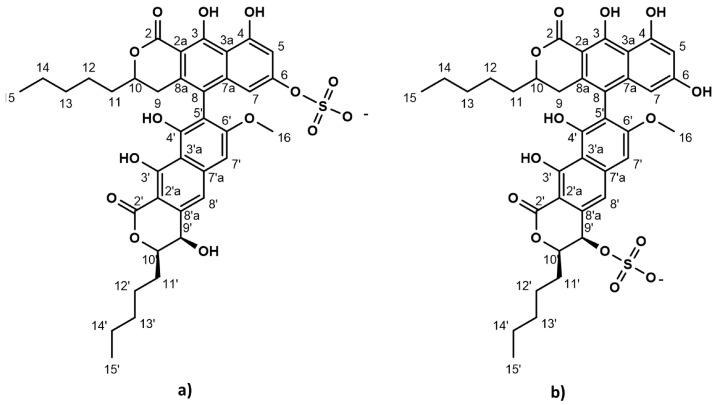
Chemical structure of lulworthinone (**a**) and acidified lulworthinone (**b**); under acidic conditions the sulphate group migrates from C6 to C9’.

The strictly marine clades of fungi are less explored in natural product discovery [8,9]. Lulworthinone was the first bioactive compound to be published from the strictly marine fungal family *Lulworthiaceae* [10]. The compound was shown to have potent activity against several clinical MRSA isolates and displayed antiproliferative activity against three human cell lines (melanoma, hepatocellular carcinoma and non-malignant lung fibroblasts) at higher concentrations. During purification, acid-induced degradation was observed, forming a structural isomer [10]. This structural isomer was identical to lulworthinone, differing only in the position of the sulphate group (Figure 1). Lulworthinone appeared to form aggregates in DMSO and methanol, which was not observed for its isomer. The compound fits structurally in the class of naphthopyrones, which have been previously isolated from different sources, including filamentous fungi. Antibacterial activity against Gram-positive bacteria has been reported for several naphthopyrones [11,12,13,14]. The wellstudied naphthopyrone viriditoxin has minimal inhibitory concentrations (MICs) in the range of 4–8 µg/mL against different *Staphylococcus* isolates, by inhibiting cell division through blocking of FtsZ polymerization [15]. Another antibacterial fungal naphthopyrone, cephalochromin, inhibits the bacterial enoyl-acyl carrier protein reductase FabI, involved in fatty acid synthesis [12].

Target identification and mode of action studies are essential steps in natural product drug discovery and development to facilitate further optimization by medicinal chemistry efforts. In this paper, the MOA of the published antibacterial natural product lulworthinone and its acidified form was investigated. The MOA was characterized using biosynthetic pathway markers, quantifying membrane permeability with a water/ion NMR detectedphospholipid vesicle permeability assay (WIND-PVPA), in vitro membrane integrity assays and membrane potential assays, time-kill curves, pharmacodynamic calculations, surface plasmon resonance (SPR), fluorescence microscopy and quantitative phase microscopy. The combined results suggest that lulworthinone is a membrane-active antibacterial compound effective against MRSA; meanwhile, its acidified form loses this ability.

## 2. Results

### 2.1. Lulworthinone Induces Transcription from Promoters Known to Respond to Cell Envelope Stress

**Figure 2:**
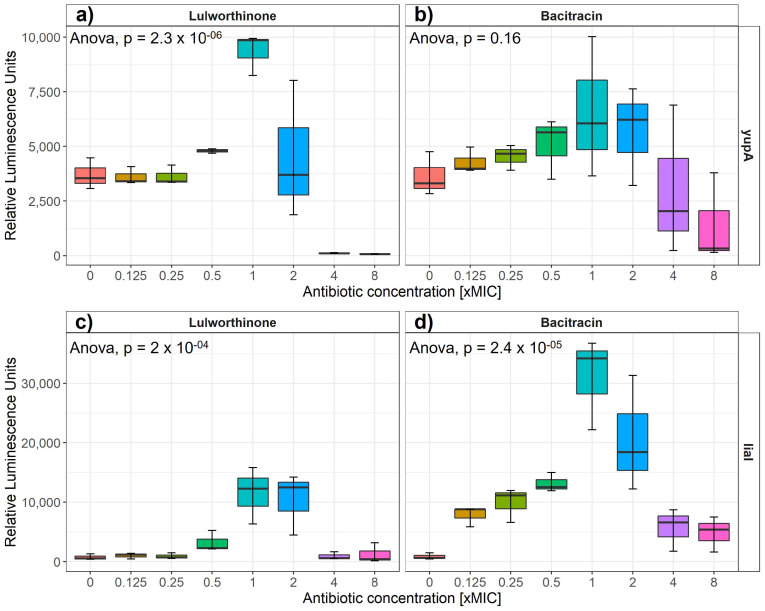
Luminesence units induced by either lulworthinone (**a**,**c**) or bacitracin (**b**,**d**) per tested concentration from 0 to 8 × MIC for *yupA* (**a**,**b**) and *liaI* (**c**,**d**) promoter fusions. Statistics performed by two-sided ANOVA comparing data of each drug concentration and biological replicates (*n* = 3).

Induction of gene expression from selected cellular pathways (i.e., DNA replication, transcription, translation, fatty acid, folic acid, cell wall and membrane) was assayed after the addition of increasing concentrations of lulworthinone. Strains of *B. subtilis* 168 containing reporter-gene constructs of relevant promoters fused to the luciferase gene are listed in Table 1. The relative luminescence activity was measured for concentrations ranging from 0 to 8 × MIC for either reference antibiotics or lulworthinone (Table 1) (Figure 2). *B. subtilis* 168 EM13 harboring the *ypuA* promoter-fusion (responding to cell wall biosynthesis inhibiton or general cell envelope stress) and *B. subtilis* 168 HMB67, carrying the *liaI* promoter-fusion (responding to general cell envelope stress) produced an increasing amount of luminescence in response to lulworthinone between 0.5 and 2 × MIC (Figure 2a,c). At 4 × and 8 × MIC, the luminescence was almost completely abolished, which indicates cell death. The control antibiotic, bacitracin, induced luciferase production at 0.125–2 × MIC from the *yupA* promoter and from the *liaI* promoter at all concentrations tested. This suggests that lulworthinone generates a general stress response in bacteria and is likely targeting the cell envelope.

### 2.2. Lulworthinone Alters Membrane Permeability without Influencing Membrane Integrity

#### 2.2.1. Lulworthinone Interacts with Membrane Lipids

SPR was used to determine the affinity of lulworthinone and its isomer towards an inert lipid bilayer composed of 1,2-dimyristoyl-sn-glycero-phosphocholine (DMPC) vesicles and its subsequent rate of dissociation (Table 2). A high partitioning of lulworthinone into lipid layers was observed with a K_P_ reaching up to 44.81 ± 2.47 × 10^3^ with a dissociation rate of 4.2 ± 0.5 × 10^−2^ s^−1^. Such values are typically encountered by very good lipid interactors (like AMC-109 [16]; see Table 2). However, there was no observable decrease in the signal (RU) after lulworthinone dissociation from the bilayer. This suggests that the lipid layer stayed intact, and that lulworthinone was able to self-aggregate on top of the lipid bilayer without disturbing it. In addition, there was no observable binding of lulworthinone to the lipid layer in concentrations < 30 µM (3 × MIC) (Figure S1). Only in higher concentrations of lulworthinone a measurable increase in resonance units was observed. Thus, the measured K_P_ for lulworthinone seems to represent both partitioning into the lipid layer and self-aggregation on top of the membrane. On the other hand, acidified lulworthinone partitioning into the lipid layer is much smaller with K_P_ − 0.76 ± 0.04 and with a much faster dissociation rate k_off_ − 5.185 ± 1.594 s^−1^. This suggests that the isomer lost its ability to bind to the lipid layer.

#### 2.2.2. Lipid Bilayer Permeability Is Not Affected by Lulworthinone

**Figure 3:**
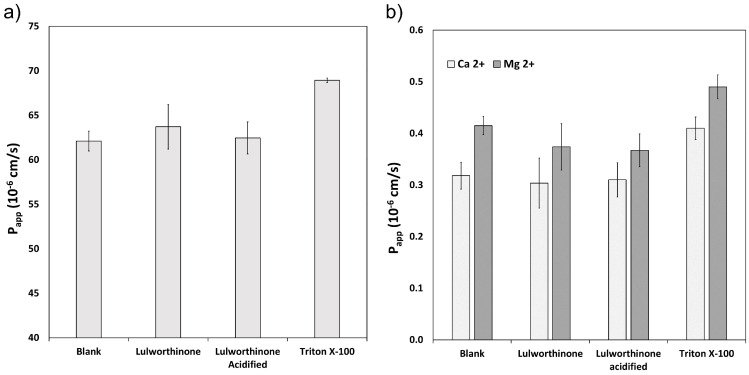
Permeability P_app_ of water (**a**) and Ca^2+^ and Mg^2+^ (**b**) measured under the influence of lulworthinone, acidified lulworthinone, and Triton X-100.

The ability of lulworthinone and its isomer to disrupt the lipid bilayer was explored using WIND-PVPA to determine the P_app_ of water and Mg^2+^ and Ca^2+^ ions across packed lipid vesicles [18]. The PVPA barriers were exposed to 100 µM of lulworthinone, acidified lulworthinone, and Triton X-100, with the latter as positive control. Figure 3 shows that neither water (Figure 3a) nor ion (Figure 3b) permeability was affected by lulworthinone. The P_app_ of Mg^2+^ in the presence of lulworthinone and the isomer were slightly lower relative to the blank (blank: 0.42 × 10^−6^ cm/s; lulworthinone: 0.37 × 10^−6^ cm/s; acidified lulworthinone: 0.37 × 10^−6^ cm/s), but these differences were not statistically relevant (*t*-test, *p* > 0.05). In comparison, the detergent Triton X-100’s higher permeability was observed for both water and ions (water: 69 × 10^−6^ cm/s; Ca^2+^: 0.41 × 10^−6^ cm/s; Mg^2+^: 0.49 × 10^−6^ cm/s). Thus, concentrations of 100 µM lulworthinone or acidified lulworthinone did not disrupt the lipid layer of membranes.

#### 2.2.3. Lulworthinone Increases the Permeability of Biological Membranes While Membrane Intergity Is Not Affected

The effect of lulworthinone on membrane integrity was investigated on bacterial cells, *B. subtilis* 168, carrying the pCSS962 plasmid from which luciferase is constitutively expressed. From this strain, bioluminescence is emitted once the bacterial cell membrane is affected, and D-luciferin from the growth medium is allowed to enter the cell. A change in membrane permeability is detected by a rise in luminescence due to substrate influx. A strong drop of luminescence is detected either after cell death or complete membrane disruption due to a fast consumption of cellular ATP needed for the enzymatic process. Bioluminescence was recorded in the presence of 0.5–4 × MIC of lulworthinone or ciprofloxacin (CIP, negative control).

**Figure 4:**
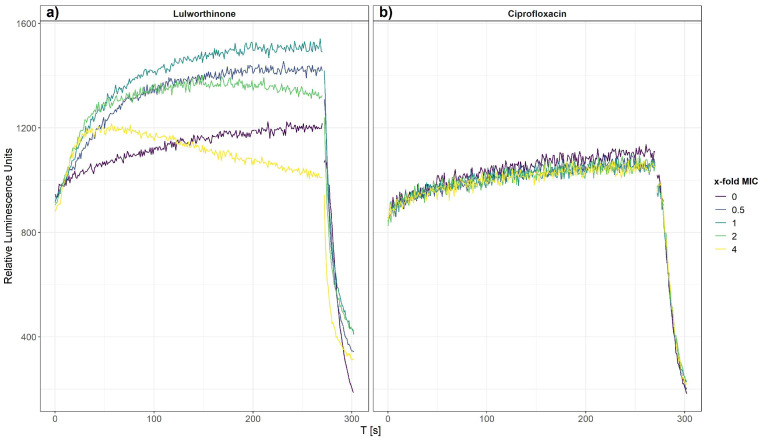
Membrane integrity of *B. subtilis* 168 carrying the pCSS962 plasmid, monitored as relative luminescence units, in the presence of different concentrations of lulworthinone (**a**) or ciprofloxacin (**b**). In both experiments, membranolytic chlorhexidine was injected at 270 s. Data presented are the means of 3 biological replicates.

After 270 s, cells that survived the first treatment were lysed by injecting a membranolytic dosage of chlorhexidine (CHX, positive control). The relative luminescence was recorded for 300 s, including the CHX injection at 270 s (Figure 4). Each concentration of lulworthinone increased the luminescence production in comparison to the basal water values (Figure 4a). The decrease in luminescence at 4 × MIC after 30 s suggests ATP depletion or cell death, as to the fast drop after CHX injection.

In contrast, CIP did not influence the membrane integrity, and the luminescence stayed at basal values of the water control until CHX injection (Figure 4b). This implies that the membrane permeability is increasingly affected by rising lulworthinone concentrations, which seemingly destroys the membrane at 4 × MIC.

### 2.3. Lulworthinone Affects the Membrane Potential

**Figure 5:**
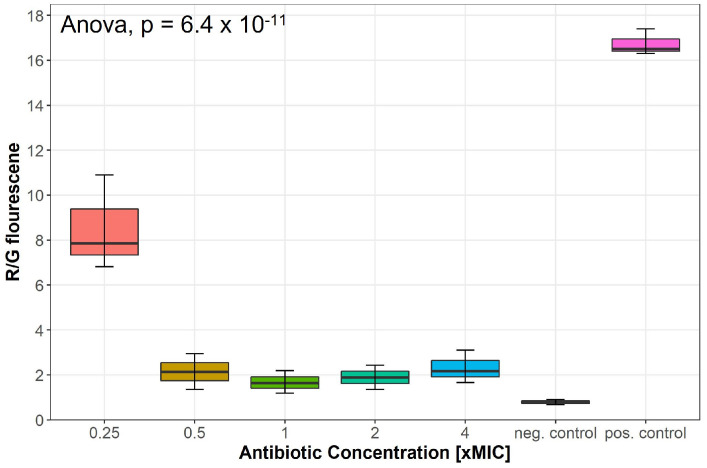
Membrane potential after exposure to increasing concentrations of lulworthinone measured by 3,3’-diethyloxa-carbocyanine iodide (DiOC_2_(3)) membrane depolarisation assay. Water (pos. control) and carbonylcyanide 3-chlorophenylhydrazone (CCCP, neg. control) were included in each assay. Statistics performed by two-sided ANOVA comparing data of each drug concentrations and biological replicates (*n* = 3).

Changes in the membrane potential after exposure to concentrations of 0.25–4 × MIC of lulworthinone was measured by a DiOC_2_(3) membrane depolarisation assay. *S. aureus* ATCC 29213 cells were stained with the membrane potential-sensitive dye 3,3’-diethyloxacarbocyanine iodide (DiOC_2_(3)) and analysed by flow cytometry. The dye fluorescence shifts from green to red by self-aggregation if the membrane potential is maintained [19]. A decrease in the ratio of red by green signals indicates a change in membrane potential. Water (positive control) and carbonylcyanide 3-chlorophenylhydrazone (CCCP, negative control) were included in each assay. At 0.25 × MIC, the membrane potential decreased by half, whereas concentrations of 0.5–4 × MIC depleted the potential close to levels of the potential inhibitor CCCP (Figure 5); an overview of all measured samples is provided in Supplementary Figure S2. This suggests that lulworthinone has a strong influence on the membrane potential.

### 2.4. Lulworthinone Influences Cell Morphology and Localization of the Cell Division Protein FtsZ

**Figure 6:**
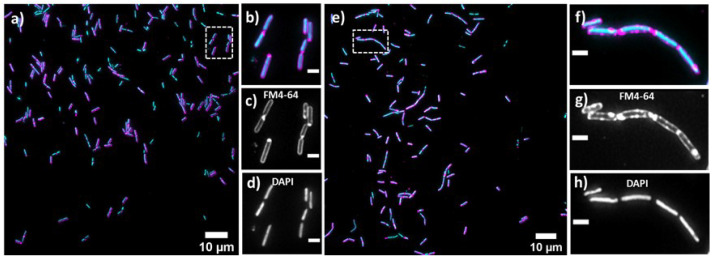
Cell morphology of *Bacillus subtilis* 168, membrane staining (FM4-64; magenta; (**c**,**d**)) and DNA staining (DAPI; blue; (**d**,**h**)) without treatment (**a**–**d**) or in the presence of 1 × MIC lulworthinone (**e**–**h**); 60× magnification in (**b**–**d**) and (**f**–**h**), respectively.

**Figure 7:**
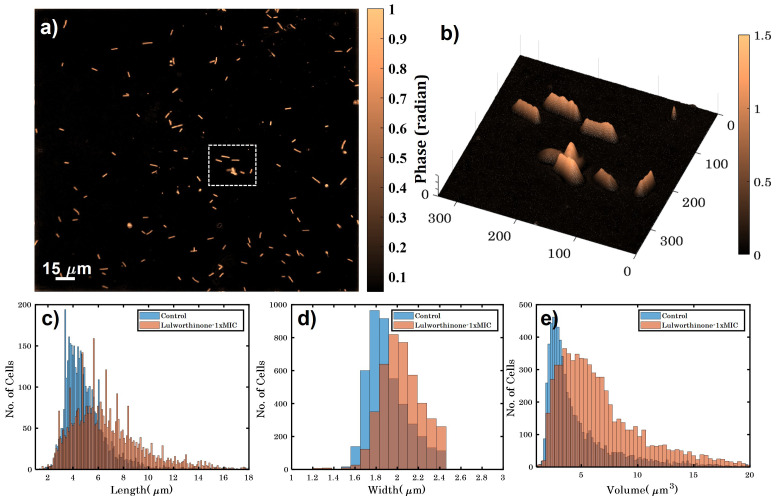
(**a**) Quantitative phase map of *B. subtilis* 168 cells (scale bar is 15 µm and color bar is in radians). (**b**) A 3D phase map of the zoomed area enclosed by white dotted box shown in (**a**). (**c**–**e**) show the variation in height, width and volume for untreated and bacteria treated with 1 × MIC lulworthinone.

**Figure 8:**
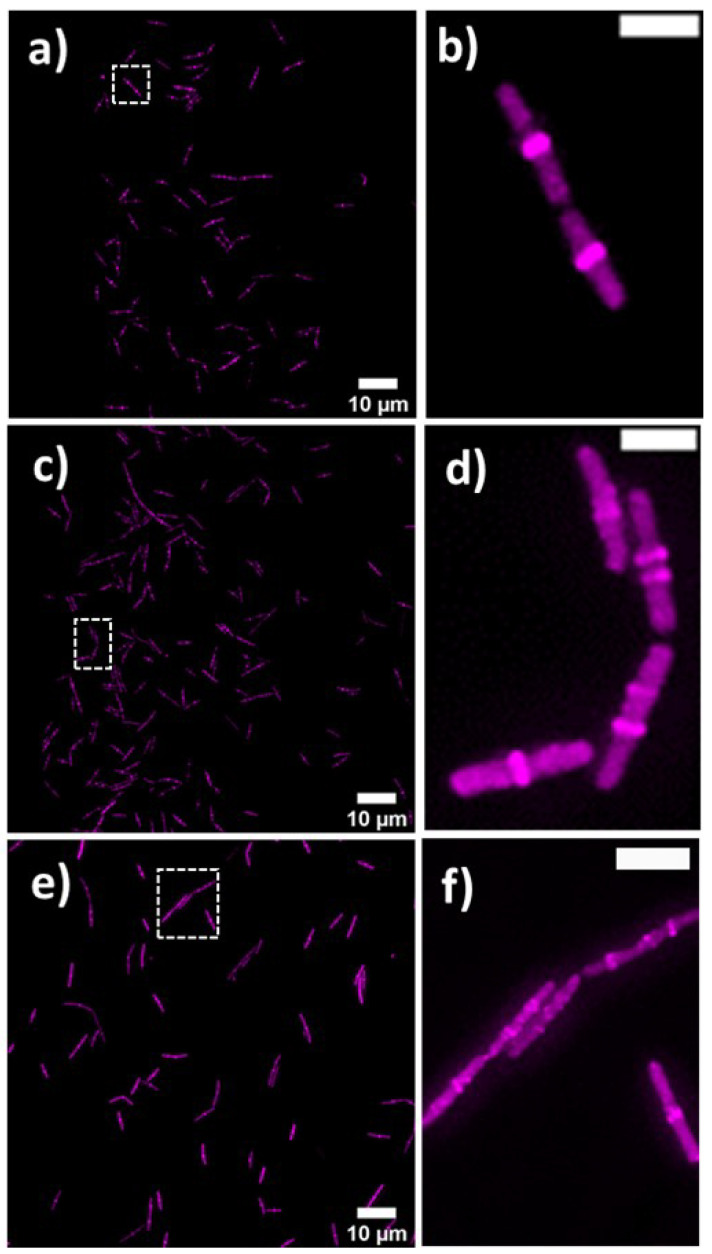
FtsZ localisation in *B. subtilis* 2020 with GFP-labeled FtsZ (**a**) without treatment, (**c**) with 1 × MIC lulworthinone or (**e**) 1 × MIC daptomycin, at 60 × magnification in (**b**,**d**,**f**), respectively.

Bacterial cell morphology in the presence of either lulworthinone or the membraneacting antibiotic daptomycin (DAP) was analysed using fluorescence microscopy. Cells were stained with membrane dye FM4-64 and DNA dye DAPI. A concentration of 1 × MIC lulworthinone affected the morphology as shown in Figure 6. When comparing the lulworthinone-treated cells (Figure 6e) to the control (Figure 6a), an increased number of bacterial filaments was observed, indicating an effect on the division process. Additionally, the altered FM4-64 distribution shown as patches of strong signal and regions of nearly no staining at all (as seen in Figure 6g) points to membrane perturbations. Changes in cell size after lulworthinone treatment were further analysed by quantitative phase microscopy (QPM). Figure 7 shows an example of a quantitative phase map (a), and the measured cell length (c), width (d) and volume (e). Data based on a total of 6700 cells from each sample, untreated or treated with 1 × MIC lulworthinone (Figure 7c–e), showed that the average cell length was extended from 4.974 to 6.763 µm, while the average width was increased from 1.898 to 2.048 µm. Accordingly, the mean volume increased from 4.788 µm${}^{3}$ to 6.649 µm${}^{3}$. Cell localisation of the cell division protein FtsZ is known to be influenced by membrane potential [20]. Thus, a reporter strain *B. subtilis* 2020 (expressing FtsZ::GFP fusion protein) was used to study the influence of lulworthinone on the membrane structure. Normally, FtsZ forms the Z-ring that defines the next septum formation and cell division site in the bacteria. The fluorescence micrographs (Figure 8) show FtsZ localisation without treatment (a,b) in the presence of lulworthinone (c,d) and with the positive control DAP (e,f). In the control (a,b), FtsZ was localized in the middle of bacteria, forming the Z-ring preceding cell division. Treatment with lulworthinone led to the elongated cells or filaments and appearance of multiple Z-rings or FtsZ patches along the cells (c,d). Daptomycin treatment (e,f) had a severe effect on FtsZ localisation and resulted in some bacteria with additional “spots” and “rings” of FtsZ. Few elongated cells and very few chains were observed. This suggests that lulworthinone has an influence on cell division, supposedly via its effect on membrane structure.

### 2.5. Lulworthinone Has a Strong Bactericidal Effect on B. subtilis

#### 2.5.1. Time-Kill Curves Reveal a Fast Bacterial Killing

**Figure 9:**
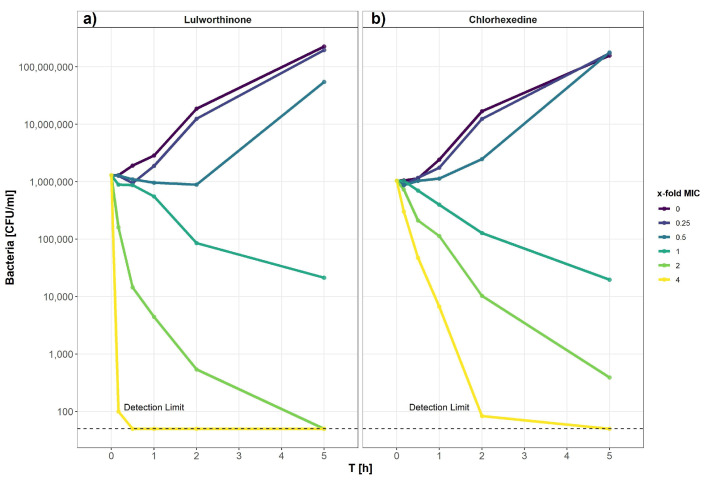
Time-kill curve of *B. subtilis* 168 of lulworthinone (**a**) and chlorhexidine (**b**).

The kill kinetics of lulworthinone was determined by measuring bacterial survival over time at multiple concentrations ranging from 0 to 64 µg/mL (0–4 × MIC) (Figure 9). Using *B. subtilis* 168, it is shown that lulworthinone (Figure 9a) was bactericidal at concentrations ≥ 1 × MIC. Higher concentrations (2–4 × MIC) led to rapid killing, and cell counts fell below the detection limit (50 CFU/mL). At 4 × MIC, this was observed within 30 min. Sub-MIC concentrations induced a lag-phase of 30 and 120 min at 0.25 and 0.5 × MIC, respectively, before growth was restored to rates comparable to the control. This suggests that some kind of adaption is required before growth continues. Time-kill curves for CHX were prepared in parallel (Figure 9b). Like lulworthinone, CHX was bactericidal above the MIC and at the highest concentration (4 × MIC), cell counts dropped below the detection limit. These data suggest that lulworthinone has a strong and fast bactericidal mode of action.

#### 2.5.2. Pharmacodynamic Calculations Reveal an Unusual Dose-Response Curve

Using the data from the time-kill curves, the pharmacodynamic parameters of lulworthinone were calculated using the *pharmacodynamic function* according to Regoes et al. (2004) [21]. The bacterial growth rates (ψ) were estimated by calculating linear regressions to the logarithm of the colony count for each concentration, respectively.

**Figure 10:**
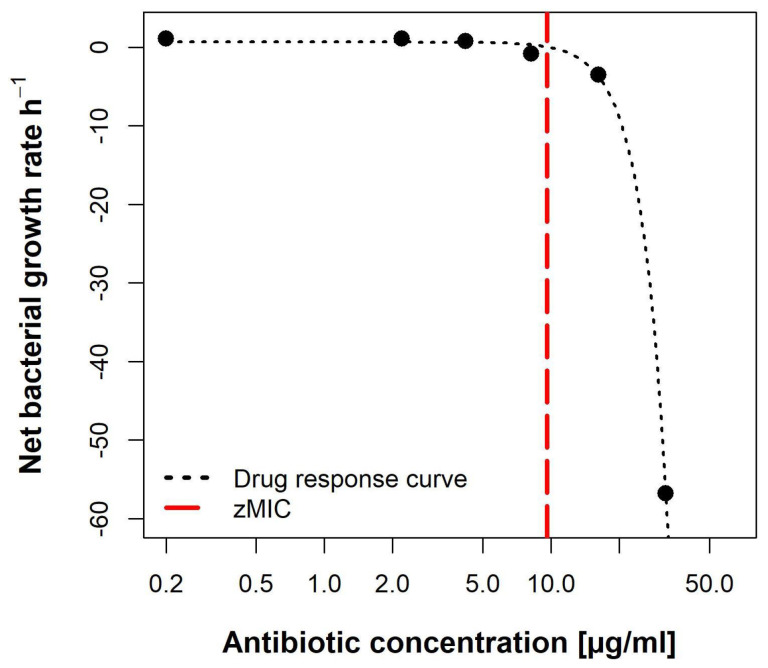
Pharmacodynamic model of lulworthinone against *B. subtilis* 168 with predicted MIC (zMIC).

The pharmacodynamic function was then fitted to the estimated ψ per concentration (Figure 10). The maximal growth rate ψ max, at 0 × MIC, was 0.6492 h^−1^. Compound lulworthinone induced a strong bactericidal effect with a minimal growth rate, at 4 × MIC, of ψ min −7.88 h^−1^. This led to a steep hill coefficent (κ) of 3.72. The estimated zMIC of 9.59 µg/mL agreed with the experimentally acquired MIC of 8 µg/mL. It was not possible to generate the typical sigmoidal “S”-shape for the drug response curve. This suggests that lulworthinone forms colloidal aggregates [22].

### 2.6. Lulworthinone Is a Self-Aggregating Molecule

#### 2.6.1. Confirmation of Aggregation

To monitor the aggregation of lulworthinone and its isomer, the molecules were assayed using dynamic light scattering (DLS). DLS is a common technique to determine particle sizes in solute by using a coherent and monochromatic source of light—a laser beam. The Brownian motion of particles causes the time-dependent fluctuation of the local concentration, which corresponds to fluctuations in the intensity of the scattering light. These fluctuations in intensity can be transformed into an autocorrelation function, from which a hydrodynamic radius can be determined using the Stokes–Einstein equation (1)

**Figure 11:**
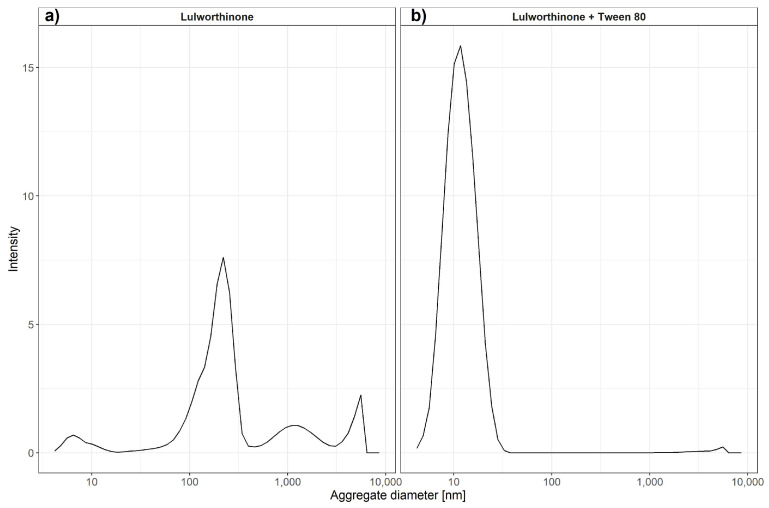
Average aggregate sizes of lulworthinone in the concentration range 0.625–320 µM in MiliQ water with 1% DMSO and without (**a**) or with 0.025% Tween 80 (**b**) measured by dynamic light scattering.

(1) R_h=kT/6πηD

where *R_h* is the hydrodynamic radius, *k* is Boltzmann’s constant, *T* is the absolute temperature, η is the shear viscosity of solvent and *D* is the translational diffusion coefficient. It has been previously shown that DLS can be used to estimate critical micellar concentrations [23]. We have used changes in intensity counts of particles > 10 nm in diameter to estimate the critical colloidal concentration, as shown in Table 3. Compound lulworthinone showed a variety of aggregates at two major diameter ranges of 192.7 ± 70.80 and 1319 ± 611.7 nm (Figure 11). To investigate if lulworthinone is a self-aggregating colloidal aggregate, we included a non-ionic detergent (Tween 80) as proposed by Ganesh et al. (2018) [24] to reverse this kind of interaction. In the presence of detergent, the aggregates vanished, and we could detect only the typical Tween 80 micelles at 10 nm, as shown in Figure 11b. This suggests that lulworthinone forms colloidal aggregates.

#### 2.6.2. The Antibacterial Activity Is Dependent on Aggregation

To determine if the antibacterial activity of lulworthinone is altered by the presence of detergent (indicating that the compound is a colloidal aggregator), Tween 80 was included in our MIC assays as proposed by Ganesh et al. (2018) [24].

The addition of detergent resulted in a strong attenuation of the antibacterial activity from 6.15 µg/mL to >128 µg/mL against *S. aureus* ATCC 25923 (Table 4). This indicates that lulworthinone antibacterial activity is based on aggregation, as the compound also lost its antimicrobial activity after acidification.

## 3. Discussion

Antibiotic resistance is making the treatment of bacterial infections difficult, and new drugs with new modes of action are needed to tackle this increasing problem. The cell membrane is a promising target for new antibiotics, as resistance is coupled to a high fitness cost for the bacterium [25]. Identifying the bacterial target and establishing the mode of action are essential steps in natural product drug discovery. This information is essential to identify promising hit compounds that can be further altered by medicinal chemistry on the road to becoming marketed drugs.

In the current study, an antibacterial compound, lulworthinone, isolated from an obligate marine fungus was studied for its MOA. The compound’s MOA includes the following key elements: (1) stress or influence on the bacterial envelope, (2) membrane permeabilization and membrane potential dissipation without destroying the membrane integrity, (3) changes in cell morphology, including increased length and width, leading to extended cells or filament formation, (4) FtsZ, a key protein for cell division, is delocalized within the bacterial cells, and (5) the antibacterial activity is based on aggregation.

As several naphthopyrones have antibacterial activity against *S. aureus* and other Gram-positive bacteria [11,12,13,14], it was not surprising to find that lulworthinone also has similar activity. This indicated that the naphthopyrone backbone might be a so-called privileged structure [26,27], with the ability to interact with a bacterial target common for some Gram-positive bacteria. The lack of activity against Gram-negative species might also be caused by the outer membrane barrier. Lulworthinone generates a general stress response in bacteria by targeting the cell envelope. The cell envelope is rather conserved among many bacterial species, and the potential for resistance development towards membrane active compounds is low as they are known to have multiple MOA targets. Taken together, this makes the cell envelope an interesting target for new antibacterial drugs (e.g., lipepopeptides (daptomycin [28]), lipoglycopeptides (teicoplanin [29]) and cyclopeptides (polymyxin B [30]). Most membrane-active molecules interact with lipophilic targets in the membrane (disrupting the lipid composition or the functional architecture), change the conformation or localisation of membrane-embedded proteins, or cause alterations in the proton motif force (PMF) [25].

However, lulworthinone does not seems to alter the structural integrity of the membrane bilayers or change the permeability of the lipid barrier. SPR indicated that lulworthinone has a high affinity towards lipids, but it also showed that there is no observable retention of lulworthinone in the lipid bilayer, as the lipid bilayer was completely recovered after the experiment. This was not expected, as good lipid associators either intercalate into the lipid bilayer and increase the overall measured signal or disrupt the layer and release vesicles and lipid matter from the surface of the chip [31]. In addition, there was no observable association of lulworthinone with DMPC vesicles at concentrations < 30 µM. Indeed, SPR results suggested that rather than disrupting lipid layer, lulworthinone can use it as a scaffold for aggregation. This fact was further confirmed by permeability results from WIND-PVPA [18]. Neither lulworthinone nor its acidified form showed any changes in water or ion transmission in artificial lipid barriers. In contrast, an increase in permeability was detectable in bacterial membranes, albeit without the loss of envelope integrity marked by cell death (as a sharp drop in fluorescence was observed only at the highest MIC concentration). The combination of these results suggests that even though lulworthinone is able to bind to the lipid bilayer, it does not disrupt artificial models, but it is still able to increase permeability in live cells. Either the disruptive effect of lulworthinone is very mild and below detection limits used in aritifical models or lulworthinone needs other membrane components present in live cells to be active.

Additionally, the dissipation of the membrane potential was detected. This can be an indication that lulworthinone interacts with surface proteins (e.g., transporters or ion channels) and inactivates them. Strahl and Hamoen (2010) [20] have shown that the membrane potential is a crucial factor for the localisation of proteins forming the cytoskeleton. Over 20 proteins involved in cell morphology, division and cell division regulation are delocalised shortly after the membrane potential is dissipated. Indeed, compound lulworthinone changed cell morphology and led to cell widening and elongation, filaments and membrane perturbation (Figure 6 and Figure 7). Signs of incomplete cell division or separation were observed.

The changes in cell morphology were accompanied by the delocalisation of FtsZ (Figure 8), a key protein for cell division as it forms the Z-ring, a molecular structure that divides cells after DNA multiplication. FtsZ was found to be delocalised into patches all over the cell or multiple Z-rings at unusual sites in the cell. As a key element for cell division, FtsZ is a focus target for antibacterial treatments [32,33,34,35,36]. As an explanation for the delocalisation, Strahl and Hamoen (2010) showed that the FtsZ guiding proteins FtsA and MinD are inactivated after loss of the membrane potential. Both have a Cterminal alpha helix structure used for membrane binding. Thus, membrane potential depletion might prevent the FtsZ guiding proteins from binding and correctly directing Z-ring formation. Without a functional Z-ring formation, cell division is affected, and filaments are formed. At sub-MIC concentrations of lulworthinone, this effect could be compensated or overcome during the observed lag phase observed for 30 and 120 min at 0.25 and 0.5 × MIC, respectively, in the time-kill curves. The current study indicates that the antibacterial activity of lulworthinone is based on self-aggregation. Compound aggregation was initially observed in the NMR experiments conducted during the structure elucidation of the compound [10]. Follow-up studies (SPR, DLS, time-kill curves, pharmacodynamics) supported the notion of aggregation. MIC testing in the presence of detergent strongly suggested that the aggregation is necessary for antibacterial activity. The structural isomer did not aggregate and was also not active against *S. aureus* 29523 (Table 3). Thus, it was concluded that lulworthinone is a colloidal aggregate, and the aggregation is necessary for its antibacterial activity. The role of aggregation in antimicrobial compounds is currently an unexplored venue as most colloidal aggregators are viewed as undesirable new drug leads due to their non-specifc protein adsorbtion and inhibition of enzymes [24,37]. To our knowledge, this is the first time that aggregation is mentioned for compounds in the napthopyrone class. However, to what extent lulworthinone is representative for the chemical class or an individual actor remains to be investigated.

## 4. Conclusions

In this study, we investigated the MOA of a dimeric naphthopyrone isolated in high yields from an obligate marine fungus. The naphthopyrone chemical class has previously been investigated for several types of bioactivities, among them antibacterial activity against Gram-positive isolates. The results from this study shows that lulworthinone exerts its activity towards the bacterial membrane without disrupting it. The membrane potential is influenced and changes in FtsZ localization, indicating an impaired cell division. Several experiments (NMR, SPR and DLS) indicate that the compound has the ability to form aggregates with itself, a property which is usually regarded as undesirable for new drug leads. To investigate if the aggregation affected the antibacterial activity, the compound’s MIC was tested in the presence of detergent. In the presence of detergent, all antibacterial activity was lost, indicating that the aggregation was necessary for the compound’s bioactivity. The study provides extended information about the target and MOA of naphthopyrones towards Gram-positive bacteria. The study also describes the effect of aggregation, and to the best of our knowledge, this is the first study in which compound aggregation has been published for naphthopyrones.

## 5. Materials and Methods

### 5.1. Bacterial Strains and Material

All bacterial strains used are listed in Table 5. Overnight cultures were grown in cationic-adjusted BD BBL Mueller Hinton II Broth (MHB II, 212322, Becton, Dickson and Company, Sparks, MD, USA) if not indicated otherwise. Lulworthinone was isolated using FLASH cromatography [10].

### 5.2. Promoter-Based Biosensor Assay

A biosensor assay was used to correlate the activity of lulworthinone with previously known MOAs. Interaction of lulworthinone with DNA replication, transcription, and translation, the cell envelope, and fatty and folic acid synthesis was determined using *B. subtilis* 168 derivates containing *luc*-genes fused to the *yorB*, *belD*, *yheI*, *yupA*, *liaI*, *fabHB*, *panB* or *liaG* promoters (Table 1). The biosensor constructs were cloned using building blocks directly from, or PCR products adapted to, the cloning enzymes used by the Bacillus BioBrick Box [40]. The plasmid pBS3Clux was used as a vector during cloning in *E.coli* Top10. The promoter regions used were either directly applied from the BioBrick Box as digestible plasmid constructs provided through the Bacillus Genetic Stock Center or adapted and amplified from Urban et al. (2007) [39] and patent US20020164602A1 by the respective primers. The promotor regions were digested with *EcoRI* and *PstI* and subsequently ligated into the vector cut with the same combination of restriction enzymes. *B. subtilis* 168 was finally transformed with the *ScaI*-linearized plasmids under 5 µg/mL chloramphenicol selection and verified by colony PCR of the disrupted *sacA* locus. Fresh colonies from agar plates were transferred to 5 mL MH medium containing 5 µg/mL chloramphenicol and incubated at 37 ${}^{\circ }$C. Overnight cultures were diluted to an OD_600_ = 0.1 and grown to an OD_600_ = 0.2 before addition to the assay plates already containing the analytes. The analytes and control antibiotics were diluted in two-fold dilution series, with the highest concentration representing 8 × of the respective MIC. A total of 5 µL of each dilution series and 45 µL bacterial suspensions were added to the wells of the 386-well plates (6007490, PerkinElmer, Waltham, MA, USA) and covered by breatheasy sealing membrane (Z380059, SIgma-Aldrich, Darmstadt, Germany) to reduce evaporation. The plates were kept in the plate reader (EnVision^(R)^, PerkinElmer, Waltham, MA, USA) at 35 ${}^{\circ }$C. The peak luminescence of the controls was compared to the luminescence of cells treated with lulworthinone. Luminescence and OD_595_ were recorded every 30 min for a total of 10 hours. The experiment was conducted three times. Data analysis and code can be found at the data repository [42].

### 5.3. Lipid Interactions Using Surface Plasmon Resonance

The SPR experiments were performed at room temperature using the T200 Biacore instrument (GE Healthcare, Chicago, IL, USA) and L1 chip. Chip treatment, cleaning, regeneration and flowrate settings are the same as in Jakubec et al. (2021) [43]. Briefly, extruded DMPC liposomes (100 nm diameter, 1 mM in 10 mM HEPES buffer pH 7.4 with 150 mM NaCl) were immobilised on a clean surface using a flowrate of 2 µL/min for 2400 s. Successful immobilisation and stabilisation was tested by an injection of 0.1 mg/mL of bovine serum albumin (BSA, A7030, Sigma-Aldrich, Saint Louis, MO, USA) for 1 min at 30 µL/min; a change of < 400 RU indicated sufficient coverage. A dilution of lulworthinone and its isomer from 4 to 128 µM in HEPES buffer was injected over immobilised vesicles. Due to the possibility of sample retention, injections were made from low to high concentration with 200 s contact time and a 400 s dissociation phase. Between runs, liposomes were regenerated by three subsequent injections of 10 mM NaOH at 30 µL/min for 30 s each. The control flow cell was treated the same way as sample cells, except 1 injection was replaced by HEPES buffer. The results were processed using in-laboratory written MATLAB scripts (MATLAB R2020a; scripts are available at https://github.com/MarJakubec, accessed on 15 March 2022). We have obtained both partitioning constant (K_P_) and dissociation rate (k_off_) using the method developed by Figueira et al. (2017) [31]. K_P_ was evaluated from the steady-state affinity at the 190-s time mark after injection and fitting the obtained curve into (Equation (2))

(2) $$\frac{RU_{S}}{RU_{L}}=\frac{\gamma_{L}K_{P}\frac{M_{S}}{M_{L}}{\left[S\right]}_{W}}{1+\sigma_{\gamma_{L}K_{P}\frac{M_{S}}{M_{L}}{\left[S\right]}_{W}}}$$

where RU_S_ and RU_L_ are the relative response of the solute (lulworthinone) and the total lipid deposition response, respectively, γL is the molar volume of the lipids, M_S_ and M_L_ are the molecular mass of the solute and lipid, respectively, and [S]_W_ is the concentration of solute in water. K_P_ and σ are obtained from fit and are, respectively, the partitioning constant and the lipid-to-solute ratio. The k_off_ rate was obtained by fitting the first 200 s of the dissociation run. We have identified the contribution of two populations to the dissociation response, which led us to use adapted formalism from Figuera et al. [31] (Equation (3)) to obtain the average k_off_ response (Equation (4)).

(3) $$S_{L}\left(t\right)=\alpha e^{-koff,\alpha^{t}}+\beta e^{-koff,\beta^{t}}+S_{L,r}$$

(4) $$k_{off}=\frac{\alpha k_{off},\alpha +\beta k_{off,\beta }}{\alpha +\beta }$$

where S_L_ is the linearised ratio of responses of the solute and lipid, which is plotted against the time of dissociation; α and β are individual populations, and S_L,r_ is the retained solute fraction.

### 5.4. Cell Membrane Integrity as Determined by Bioluminescence

A bioluminescence-based assay developed by Virta et al. (1995) [38] was used to investigate the membrane disruptive properties of lulworthinone. Upon the disruption of the membrane, the intracellular produced luciferase would interact with its extracellular provided substrate—D-luciferin—and emit luminescence in real time. For this, a *Bacillus subtilis* 168 strain expressing luciferase encoded on the pCSS962 plasmid was used. Concentrations ranging from 0 to 4 × MIC, including chlorhexidine as a membranolytic control (200 µg/mL) and ciprofloxacin as a non-membrane active negative control, were tested. Overnight cultures were grown in MHB II containing 5 µg/mL chloramphenicol (220551, Calbiochem, San Diego, CA, USA). The bacteria were pelleted and resuspended in fresh MHB II to OD_600_ of 0.1 D-luciferin potassium salt (pH 7.4, SynChem Inc., Elk Grove Village, IL, USA), which was added to achieve a final concentration of 1 mM. Subsequently, 96-well plates (655209, Greiner Bio-One, Kresmmuenster, Austria) containing 20 µL of compound dilutions were prepared and loaded into a plate reader (Synergy H1 Hybrid reader, BioTek, Winooski, VT, USA). For each test well, 180 µL bacterial inoculums were injected by an automatic injector. The bioluminescence was measured for 270 s before 35 µL chlorhexidine (vnr 007214, Fresenius Kabi Norge AS, Halden, Norway) was added at a membranolytic concentration (30 µg/mL). The luminescence was measured for an additional 30 s. The light emission with CHX indicates the lysis of bacterial cells that are still alive after the first treatment. The experiment was performed three times. Data, analysis and code at can be found in the data repository [42].

### 5.5. DiOC_2_(3) Cytoplasmic Membrane Depolarization Assay

To characterize the influence of lulworthinone on the cytoplasmic membrane potential, the fluorescence of a membrane potential indicator dye was measured with flow cytometry. The *Bac*Light Bacterial Membrane Potential Kit (B34950, Invitrogen, Carlsbad, CA, USA), which includes a fluorescent membrane potential indicator dye, 3,3’-Diethyloxacarbocyanine iodide (DiOC_2_(3)) and carbonyl cyanide m-chlorophenyl hydrazone (CCCP) as a membrane potential inhibitor [19], was used. In low abundance, DiOC_2_(3) emits green fluorescence in bacterial cells. When cells maintain their membrane potential, they accumulate more dye, which self-associates, and the fluorescence shifts into the red spectrum. The assay was performed according to the manufacturer. *B. subtilis* 168 was replaced by *S. aureus* ATCC 29213, since it showed much clearer detectable differences in potential change. In short, an inoculum of 1 × 10^6^ CFU/mL was prepared in sterile filtered (0.22 µm pore size) PBS (P4417, Sigma-Adrich, Saint Louis, MO, USA). For each sample, 1 mL inoculum was transferred in flow cytometer tubes (352054, Corning Science, Reynosa, Mexico). Additional tubes for a depolarized control (CCCP, 10 µL of 500 µM stock) and unstained control were included. Lulworthinone was added for concentrations ranging from 0.25 to 4 × MIC. Samples were vortexed and added to 10 µL of DiOC_2_(3) (to each tube besides the unstained control), mixed and incubated for 30 min. Samples were exited at 480 nm, and fluorescence was collected with 530/30 nm and 616/23 nm emission filters using the BD LSRFortessa Cell Analyser (647794, BD Bioscience, Eysins, Switzerland). Samples were gated on the bacterial cell size with a set threshold at 1500 sideward scatter. A total of 10,000 events were collected. The data were analysed using the FlowJoe software (v10.8.0, FlowJo, LLC, Ashland, OR, USA), and the gated population mean fluorescence intensity (MFI) was obtained in a red vs. green fluorescence dot plot. The ratio of red MFI divided by green MFI reflecting the membrane potential. The experiment was performed three times; data, analysis, and code can be found in the data repository [42].

### 5.6. Cell Morphology and Biomarker Detection Using Microscopy

*B. subtilis* 168 was grown in MHB II at 37 ${}^{\circ }$C under agitation. Reporter strain 2020 was grown in MHB II supplemented with 100 µg/mL spectinomycin (S9007, Sigma-Aldrich, Saint Louis, MO, USA) and 0.5% xylose (PHR2102-500MG, Merck Ag, Darmstadt, Germany) at 30 ${}^{\circ }$C under agitation. Additionally, MHB II was supplemented with 1.25 mM CaCl_2_ for all experiments with daptomycin (DAP, Cubicin, Novartis, London, UK) [44]. For *B. subtilis* 168, aliquots from the overnight cultures were diluted 1:50 in prewarmed MHB II and incubated at 37 ${}^{\circ }$C under agitation until an OD_600_ of 0.3. The cultures were diluted 1:1 with the solutions of lulworthinone and the reference antibiotic DAP in the wells of a 96-well microtiter plate (249943 Nunc, Thermo scientific, Loughborough, UK). The final concentration of all compounds in the wells was 1× MIC. In parallel, a 1:1 combination of the cultures with sterile Milli-Q H_2_O or 1.25 mM CaCl_2_ for DAP were used as untreated controls. Bacteria were incubated for 90 min at 37 ${}^{\circ }$C with agitation and pelleted at 13.5× *g* for 5 min and carefully suspended in prewarmed 0.9% NaCl. Subsequently, bacteria were stained with 12 µg/mL FM 4-64 (T13320, Invitrogen, Waltham, MA, USA) and 2 µg/mL DAPI (D9542, Sigma-Aldrich, Saint Louis, MO, USA) for 25 min at 37 ${}^{\circ }$C with agitation. Cells were pelleted again and carefully resuspended in preheated 0.9% NaCl. Aliquots of the bacterial suspensions were applied to the bottom of 35 mm confocal dishes (75856-742, VWR, Radnor, PA, USA) and covered by 2.4% agarose pads prepared in 0.9% NaCl. For *B. subtilis* 2020, the sample preparation was like the one described above, with the following modifications. Aliquots from the overnight cultures were incubated in the presence of 0.5% xylose. Samples were treated for a total of 45 min prior to microscopy. No washing steps were included. Incubation at all steps was performed at 30 ${}^{\circ }$C with agitation. Aliquots of the stained suspensions were applied to the round 1.5 coverslips (631-0161, VWR, Radnor, PA, USA). The fluorescence images of the bacteria were acquired via a DeltaVision Elite Deconvolution Microscope (GE Healthcare, Chicago, IL, USA). For the wide-field deconvolution imaging of bacteria, an oil immersion 60 × (1.42NA) objective lens was utilized. For DAPI, the excitation wavelength range was 381–401 nm, and the emission was in the 409–456 nm range. The excitation and emission wavelength range for FM 4–64 were 425–495 nm and 652–700 nm, respectively. For GFP, the excitation and emission wavelength range were 425–495 nm and 500–550 nm, respectively. To achieve a superior contrast and resolution in images, a volume stack of 12 planes over 3 µM depth were acquired and deconvolved. For each treatment, 10–20 imaging fields were viewed. Experiments were done in three biological replicates. Pictures can be found at the data repository [42].

### 5.7. Cell Morphology Determination with Quantitative Phase Microscopy

Digital holography-based quantitative phase microscopy (QPM) has been developed to obtain quantitative information about the bacteria in a label-free manner. QPM improves the image contrast of transparent cells while quantifying parameters such as: optical thickness (sample thickness x refractive index (n)), refractive index variation, cell dry mass and other morphological parameters [45,46]. *B. subtilis* 168 were cultivated in MHB II at 37 ${}^{\circ }$C until an OD_600_ = 0.3 was reached. The cultures were diluted 1:1 with the solutions of lulworthinone for 90 min. Subsequently, 90 µL samples were pelleted at 13.5× *g* for 5 min and carefully suspended in 200 µL PHEM (pH 7.3) buffer containing 2% paraformaldehyde (PFA) and 1% glutaraldehyde (GA). For QPM measurements, the bacterial cells were placed in a polydimethylsiloxane (PDMS) chamber on a reflective Si substrate and covered with a standard 1.5 thickness coverslip. Before sample preparation, the surface of the Si substrate was treated with 0.1% poly-L-lysin for 10 min to enhance cell attachment. The interferograms were acquired with a 60 × (1.2NA) objective lens and further post-processed in MATLAB to get the phase map of the bacteria. The individual bacteria were segmented for the quantitative assessment of length, width, volume and other morphological parameters of the bacteria.

### 5.8. Kill Kinetics Using Time-Kill Curves

The kill kinetics of lulworthinone can be expressed as rate over time with a fixed drug concentration—so called time-kill curves [47]. Time-kill curve analyses were performed by culturing *B. subtilis* 168 in MHB II at antimicrobial concentrations ranging from 4 × MIC to 0.25 × MIC. The MICs were determined according to CLSI guidelines [48], presented in Table 5). The antimicrobials examined were lulworthinone and chlorhexidine (17850, Sigma-Adrich, Saint Louis, MO, USA). Cultures were inoculated from MH agar plates and grown in MHB II for 18–20 h at 37 ${}^{\circ }$C, reinoculated and grown to mid-log phase for 3 h in MHB II, before diluting them to 1 × 10^6^ CFU/mL in pre-warmed MHB II (37 ${}^{\circ }$C). For the test setup, the two-fold drug concentrations were prepared in 750 µL MHB II each. An antibiotic-free growth control was included and prepared in a 24-well polypropylene plate (SKU:1300-00312, Bellco Glass Inc., Vineland, NJ, USA). For each drug concentration, 750 µL inoculum was added to each well. The plates were incubated for 5 h at 37 ${}^{\circ }$C and sampled at 10, 30, 60, 120 and 300 min. Samples for the start time point (T_0_) were taken from the inoculum, diluted 1:1 with MHB II. Each sample was diluted seven times in PBS, and 20 µL of each dilution was plated out in a run-streak on MH agar plates. Samples were plated in duplicates; each experiment was performed three times. Data, analysis and code can be found in the data repository [42].

### 5.9. Pharmacodynamic Parameters

The data of the time-kill curves were used to model the pharmacodynamic parameters of lulworthinone. The bacterial net growth rates (ψ) were estimated from the surviving bacteria (CFU/mL) over time between 0 and 300 min, as described above. The pharmacodynamic function [21] was fitted to the ψ present at different drug concentrations. In this model, the top asymptote ($\psi_{max}$) and the bottom asymptote ($\psi_{min}$) indicate the maximal and minimal bacterial net growth rate in relation to the drug concentration. The slope of the curve (κ or the Hill coefficient) represents the relationship between bacterial growth and antimicrobial concentration. The antimicrobial concentration that results in a ψ of zero is the pharmacodynamic MIC (zMIC). Data analysis was done in R [49], and the *censReg* package [50] was used to calculate concentrations containing censored data points. Data and code are available at the data repository [42].

### 5.10. Aggregation Formation Detection with Dynamic Light Scattering

We have tested the ability of lulworthinone to form oligomers by Zetasizer Nano ZS (Malvern Ins., Malvern, UK). Lulworthinone was dissolved in 5% DMSO in MiliQ and then diluted to obtain a concentration range from 320 µM to 0.625 µM in 1% DMSO. We have tested its ability to form oligomers at 37 ${}^{\circ }$C with or without the presence of 0.025% Tween 80.

### 5.11. Influence of Detergent on Antibacterial Activity

To determine if lulworthinone forms colloidal aggregates that affect its antimicrobial activity, an MIC assay including a non-ionic detergent was used. The antibacterial activity of a colloidal aggregate should be heavily attenuated in the presence of non-ionic detergents [24,51]. An MIC assay was performed according to CLSI guidelines [48] using *S. aureus* ATCC 25923. The MIC values used are from the previous study [10]. Overnight cultures were grown in MHB (275730, BD Difco, Le Pont de Claix, France) at 37 ${}^{\circ }$C. Two-fold dilution series of lulworthinone ranging from 128 µg/mL to 0.25 µg/mL with or without 0.025% (*v/v*) Tween 80 (P8074, Sigma-Aldrich, Saint Louis, MO, USA) were tested.

Assay was conducted in 96-well plates (Nunclon Δ 734-2073, VWR, Radnor, PA, USA). OD_600_ values were recorded by a plate reader (Victor multilabel counter, PerkinElmer, MA, USA) at 37 ${}^{\circ }$C for 24 h. Each test run included a growth control (media and inoculum) and a sterility control (media and water), and for quality assurance, *S. aureus* ATCC 25923 was tested against gentamicin (A2712, VWR, Radnor, PA, USA). Tests were performed in triplicates with three technical replicates; median MIC values are displayed.

### 5.12. Data Analysis

Data handling, analysis, statistics and presentation were done using R 4.1.0 [49], the *tidyverse* package [52], the *ggplot2* package [53], the *ggpubr* package [54] and the *cowplot* package [55]. Data documentation was done using the *bookdown* package [56].

## Figures and Tables

**Table 1:** Bacterial strains sensing stress on key molecular pathways.

					MIC in µg/mL	
Bacteria	Strain Number	Target Pathway	Promotor	Control Antibiotic	Control Antibiotic	Lulworthinone
*Bacillus subtilis* 168	EM10	DNA replication	*yorB*	Ciprofloxacin	0.031	8
*B. subtilis* 168	EM11	Transcription	*belD*	Rifampicin	0.5	8
*B. subtilis* 168	EM12	Translation	*yheI*	Erythromycin	0.125	8
*B. subtilis* 168	EM13	Cell wall and membrane	*yupA*	Bacitracin	16	8
*B. subtilis* 168	HMB62	Viability control	*laiG*	All antibiotics	*	8
*B. subtilis* 168	HMB67	Cell wall and membrane	*liaI*	Bacitracin	16	8
*B. subtilis* 168	HMB69	Fatty acid synthesis	*fabJB*	Triclosan	4	8
*B. subtilis* 168	HMB70	Folic acid synthesis	*panB*	Trimethoprim	1	8

Abbreviations: MIC—minimal inhibitory concentration; * MICs are equivalent to the other strains.

**Table 2:** Lulworthinone and acidified lulworthinone’s affinity towards and subsequent dissociation rate from an inert lipid bilayer. Positive and negative [17] controls are included.

Treatment	$K_{P}$ $\times 10^{3}$	$k_{off}$ $s^{-1}$
Lulworthinone	44.81 ± 2.47	0.042 ± 0.005
acid. Lulworthinone	0.76 ± 0.04	5.185 ± 1.594
pos. control—AMC 109	14.97 ± 0.99	0.174 ± 0.007
neg. control—LWwNKr	0.40 ± 0.02	1.746 ± 0.162

*K_P_*—partitioning constant, *k_off_*—dissociation rate.

**Table 3:** Aggregate sizes determined by DLS.

Treatment	Environment	Critical Aggregation Concentration (CAC)	Prevalent Size of Aggregates at CAC
Lulworthinone	37 ${}^{\circ }$C	53.71 µM	117.4 ± 25.9 d.nm
Lulworthinone with 0.025% Tween 80	37 ${}^{\circ }$C	No aggregation	No aggregation

**Table 4:** Antibacterial activity of lulworthinone.

Bacterial Strain	Treatment	MIC
*Staphylococcus aureus* ATCC 25923	Lulworthinone	6.15 µg/mL
*S. aureus* ATCC 25923	Lulworthinone + Tween 80	>128 µg/mL
*S. aureus* ATCC 25923	Acidified lulworthinone	>128 µg/mL

**Table 5:** Bacterial strains.

		MIC in µg/mL					
Strain	Relevant Characteristics	Lulworthinone	Acid. Lulworthinone	CHX	CIP	DAP	References
*Bacillus subtilis* 168	-	8	-	0.5	-	-	ATCC 23857
*B. subtilis* 168	pCSS962	8	-	0.5	0.00195	-	[38]
*B. subtilis* 168 EM10	P${}_{yorB}luxABCDE$	8	-	-	-	-	[39,40,41]
*B. subtilis* 168 EM11	P${}_{belD}luxABCDE$	8	-	-	-	-	[39,40,41]
*B. subtilis* 168 EM12	P${}_{yheI}luxABCDE$	8	-	-	-	-	[39,40,41]
*B. subtilis* 168 EM13	P${}_{yupA}luxABCDE$	8	-	-	-	-	[39,40,41]
*B. subtilis* 168 HMB62	P${}_{liaG}luxABCDE$	8	-	-	-	-	[39,40,41]
*B. subtilis* 168 HMB67	P${}_{liaI}luxABCDE$	8	-	-	-	-	[39,40,41]
*B. subtilis* 168 HMB69	P${}_{fabHB}luxABCDE$	8	-	-	-	-	[39,40,41]
*B. subtilis* 168 HMB70	P${}_{panB}luxABCDE$	8	-	-	-	-	[39,40,41]
*B. subtilis* 2020	*amyE::spc Pxyl-gfp-ftsZ*	-	-	-	-	2	[20]
*Echerichia coli* Top10	*pBS3Clux*	-	-	-	-	-	[39,40]
*Staphylococcus aureus* 29213	-	6.25	-	-	-	-	ATCC 29213
*S. aureus* 25923	-	6.25	>128	-	-	-	ATCC 25923

Abbreviations: MIC—minimal inhibitory concentration; CHX—chlorhexedine; CIP—ciprofloxacin; DAP—daptomycin.

## Data Availability

The data presented in this study are openly available in DataverseNO at https://doi.org/10.18710/6Z0VJX, accessed on 15 March 2022.

## Supplementary Materials

The following are available online at https://www.mdpi.com/article/10.3390/md20050277/s1, Figure S1: SPR sensogram for (A) lulworthinone and (B) acidified lulworthinone; Figure S2: Membrane potential shift in the presence of lulworthinone.
