# Supplementary material for: Lulworthinone: In Vitro Mode of Action Investigation of an Antibacterial Dimeric Naphthopyrone Isolated from a Marine Fungus
Source: Mar Drugs. 2022 Apr 21;20(5):277. doi: 10.3390/md20050277 (PMC9147123; doi:10.3390/md20050277)
Supplement: Supplementary file 1 [file marinedrugs-20-00277-s001.zip › marinedrugs-1660465-supplementary.pdf]

A

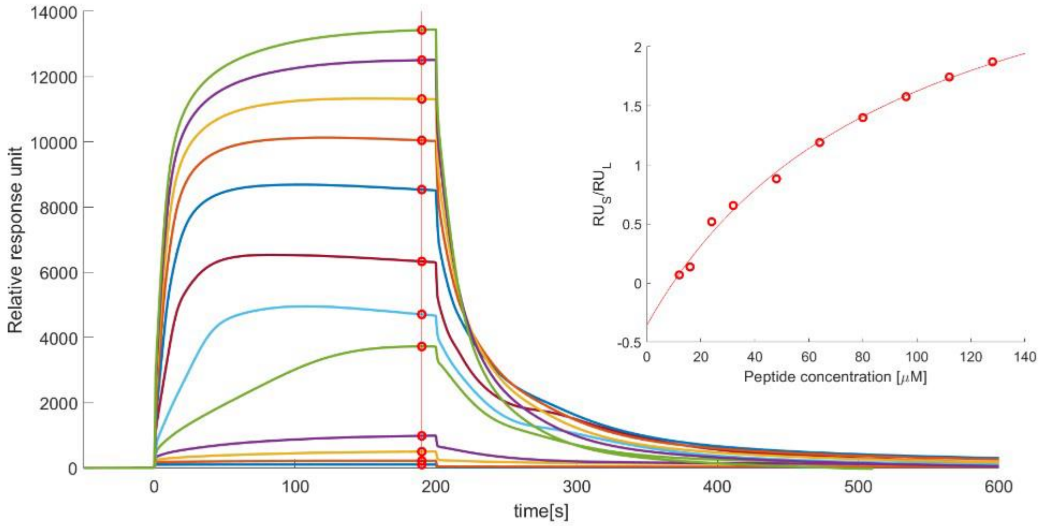

B

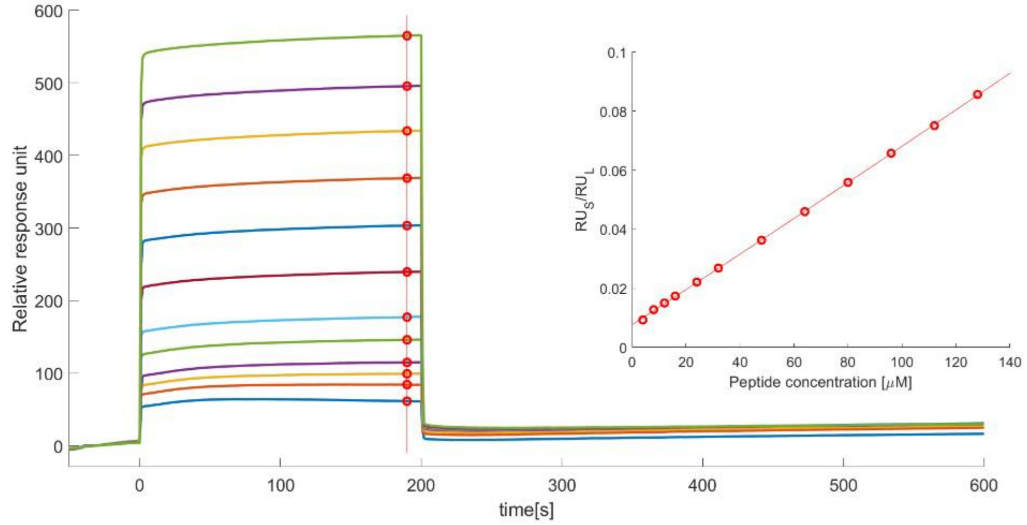

Figure S1. SPR sensogram for (**A**) lulworthinone and (**B**) acidified lulworthinone. Red line points to a steady state where relative response was read. Values were fitted (inset) to obtain KP. Please note the different range in relative response units for both compounds.

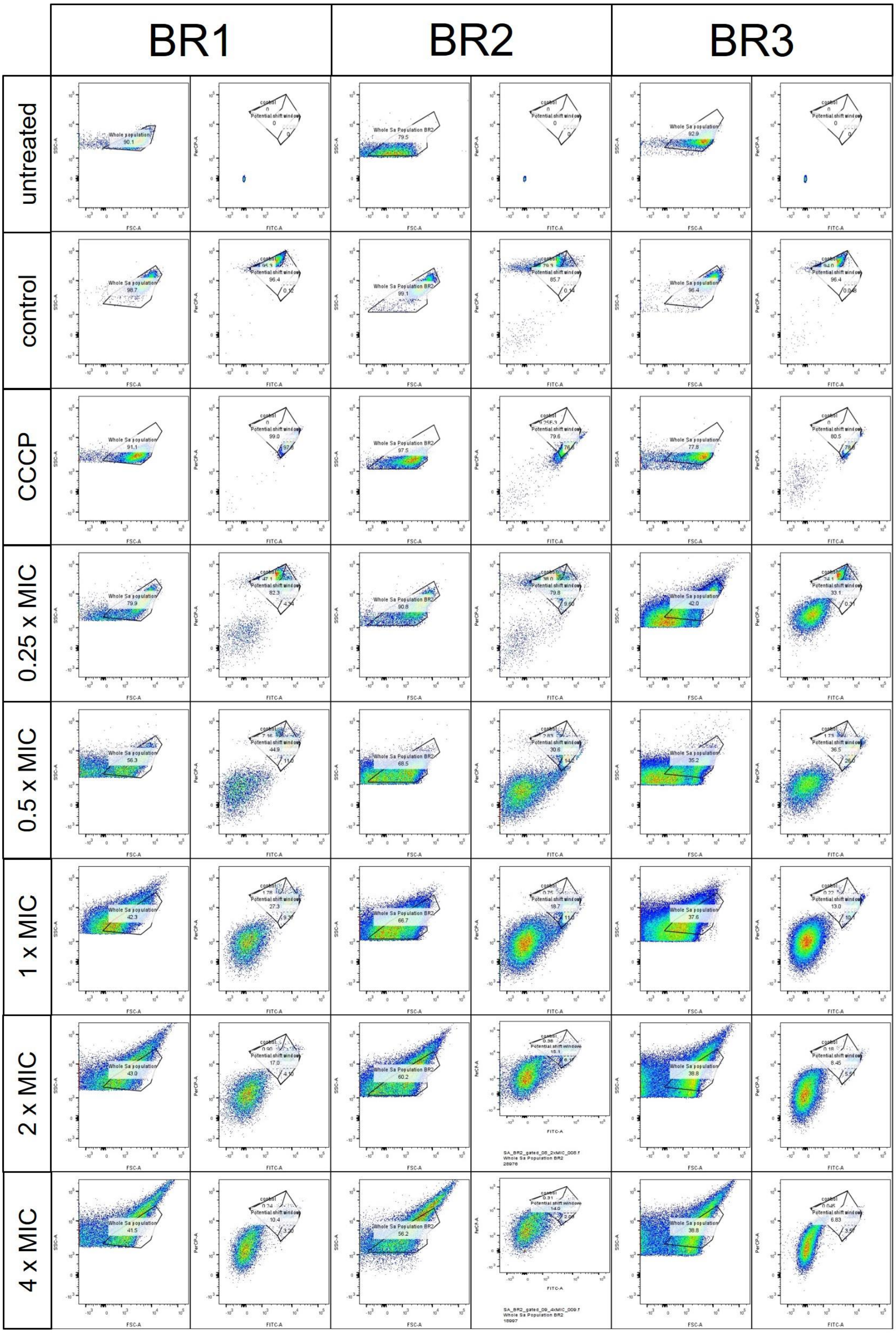

Figure S2. Membrane potential shift in the presence of lulworthinone. Red cultures have a membrane potential, while green cultures show a dissipation the membrane potential.
